# Will Ion Channels Help Coccolithophores Adapt to Ocean Acidification?

**DOI:** 10.1371/journal.pbio.1001087

**Published:** 2011-06-21

**Authors:** Robin Mejia

**Affiliations:** Freelance Science Writer, Albany, California, United States of America

**Figure:**
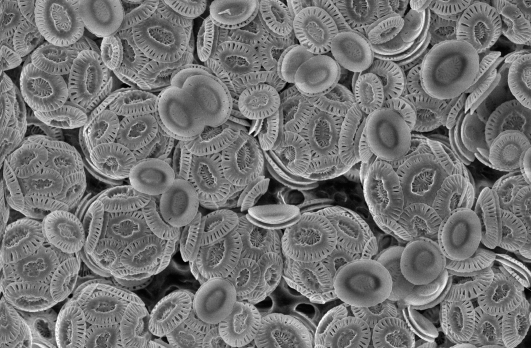
Coccolithophore cells covered with calcium carbonate (chalk) scales. Progress in understanding the unique physiology of these globally important organisms will help us to understand how they may respond to changing ocean chemistry. Image courtesy Dr. Alison Taylor.

Phytoplankton come in a wide variety of shapes, sizes, and taxa, but the single-celled coccolithophores are among the most unusual. While most plankton congregate near upwellings of nutrient-rich water from the ocean floor, coccolithophores can also thrive in nutrient-poor waters. Widely dispersed throughout the oceans, these aquatic algae play an important, if not completely understood, role in carbon deposition and marine geochemical cycling and can create massive blooms visible from the sky. Their name derives from their beautiful, tiny, calcium carbonate–based scales, called coccoliths. These scales form a coat around the cells and are often produced in excess. When cells die or when blooms naturally collapse, the scales sink to the ocean floor.

Unlike other marine organisms that create calcium carbonate structures, like coral, coccolithophores produce their scales in an intracellular compartment and then secrete them to the surface. The internal calification process requires calcium and inorganic carbon as inputs. Studies indicate the algae rely on HCO3^−^ as a carbon source for calcification, and the process of scale-building releases a mole of H^+^ inside the cell for every mole of calcium carbonate that is precipitated. In this issue of *PLoS Biology*, Alison Taylor and colleagues describe coccolithophore ion channels that allow H^+^ to diffuse out of the cell, showing how the cells avoid acidification. They further suggest that H^+^-transporting ion channels may be much more widely distributed than previously believed.

Using published studies of calcification rates in coccolithophores, the researchers calculated that the amount of H^+^ produced by the internal creation of calcium carbonate shells would cause rapid acidification of the cell if it were not removed. A number of processes, including photosynthesis, could remove some amount of H^+^; however, no mechanism that had been studied appeared to account for the rapid removal (or sequestration) that would be needed to avoid cellular acidosis.

Ion channels, which could facilitate that level of H^+^ transfer, do occur in the membranes of mammalian cells. To determine whether such channels were at work in the phytoplankton, the researchers carried out patch clamp recordings of cellular membranes of *Coccolithus pelagicus spp braarudii*. They found not just H^+^-permeable ion channels but also rates of H^+^ transfer that were adequate to explain why the cells don't acidify when they produce their scales. To verify that the ion channels were responsible for the H^+^ transfer, they inserted the gene for the coccolithophore H^+^ channel into human cell lines, and found that the transgenic human cells showed increased H^+^ transfer as well.

The H^+^ channel appears to enable the phytoplankton avoid acidosis, and its functioning may in part determine the species' ability to adapt to ocean acidification.

Over the past century, as carbon dioxide levels have increased in the earth's atmosphere, the earth's oceans have absorbed about half of that carbon. This process helps to naturally mitigate the effects of carbon dioxide in the atmosphere; however, as a result, the pH of ocean waters, at least at the surface, has begun to decline. Ocean acidification will likely affect many species, but calcifiers are believed to be some of the most vulnerable, as increasing acidity can lead to demineralization of calcium carbonate shells.

Taylor and colleagues showed further that the ability to calcify was dependent on the cell's ability to regulate intracellular pH. Treatments that disrupted the transport of H^+^ across the membrane also disrupted intracelluar pH and calcification.

After determining that coccolithophore ion channels functioned similarly to their mammalian counterparts, the researchers compared their genes. They describe coccolithophore and mammalian H^+^ channels as showing a “weak similarity” at the genetic level, but maintaining similar organization and structure, which they suggest indicates a common ancient ancestry. They then scanned the genomes of other organisms, and found copies of coccolithophore-like H^+^ channel genes in other species, including diatoms and social amoebas, in which they had not been previously identified or studied. This suggests, they say, that the channels are widely distributed and likely play an important role in the physiology of many eukaryotes.

**Taylor AR, Chrachri A, Wheeler G, Goddard H, Brownlee C (2011) A Voltage-Gated H^+^ Channel Underlying pH Homeostasis in Calcifying Coccolithophores. doi:10.1371/journal.pbio.1001085**

